# Supplementary material for: 1,2,4-Oxadiazole-Based Bio-Isosteres of Benzamides: Synthesis, Biological Activity and Toxicity to Zebrafish Embryo
Source: Int J Mol Sci. 2021 Feb 27;22(5):2367. doi: 10.3390/ijms22052367 (PMC7956408; doi:10.3390/ijms22052367)
Supplement: Supplementary file 1 [file ijms-22-02367-s001.pdf]

Supporting information

1,2,4-Oxadiazole Based Bio-isosteres of Benzamides: Synthesis, Biological Activity and Toxicity to *Zebrafish Embryo*

Sen Yang, Chaoli Ren, Tianyang Ma, Wenqian Zou, Li Dai, Xiaoyu Tian, Xinghai Liu, Chengxia Tan *

College of Chemical Engineering, Zhejiang University of Technology, Hangzhou 310014, China;

* Corresponding author.
E-mail address: tanchengxia@zjut.edu.cn

The ¹H NMR , ¹³C NMR and HRMS spectra of the representative title compounds.

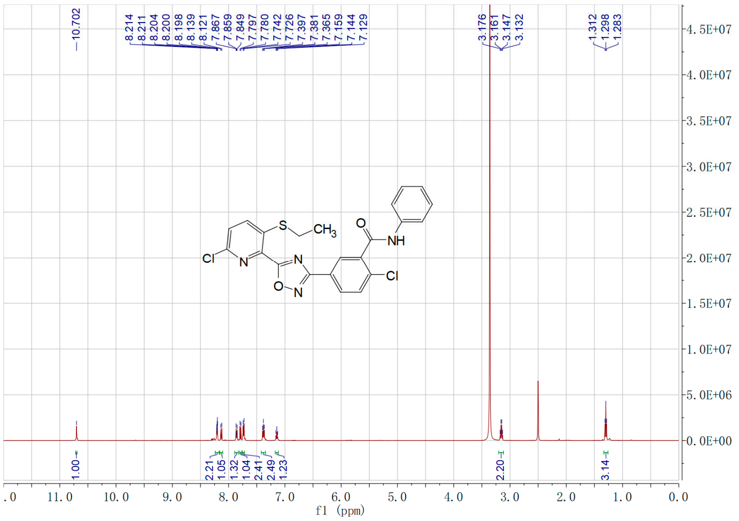

Fig. S1. The ¹H NMR spectrum of compound **9a**

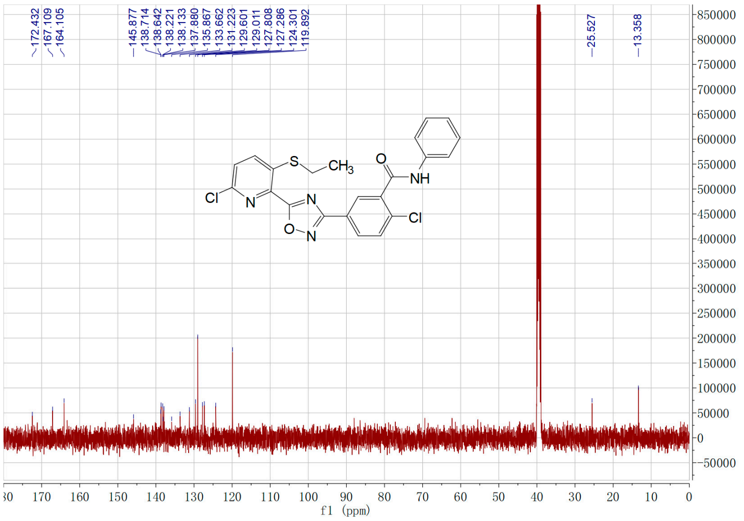

Fig. S2. The ¹³C NMR spectrum of compound **9a**

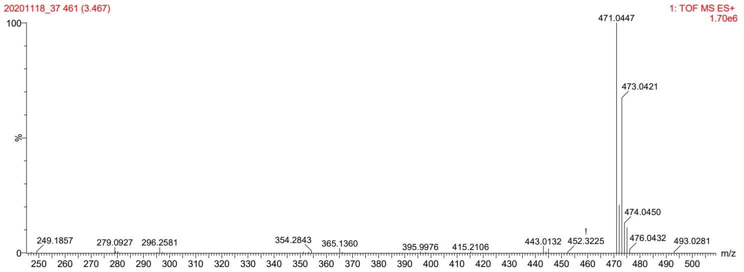

Fig. S3. The HRMS spectrum of compound **9a**

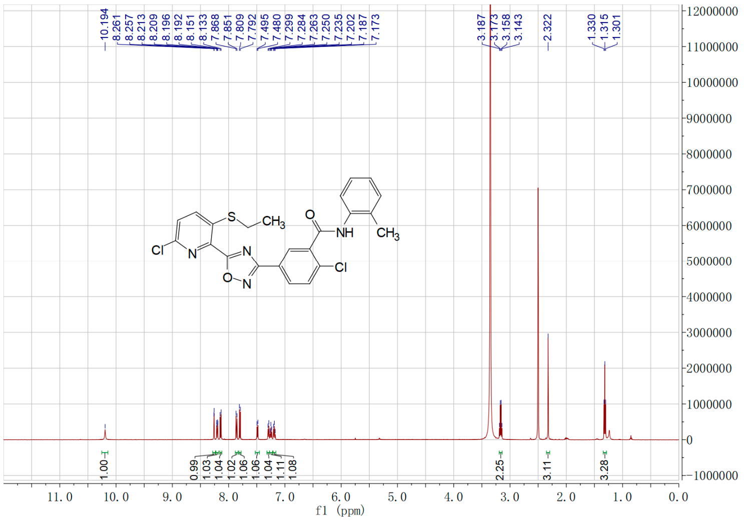

Fig. S4. The ^{1}H NMR spectrum of compound **9b**

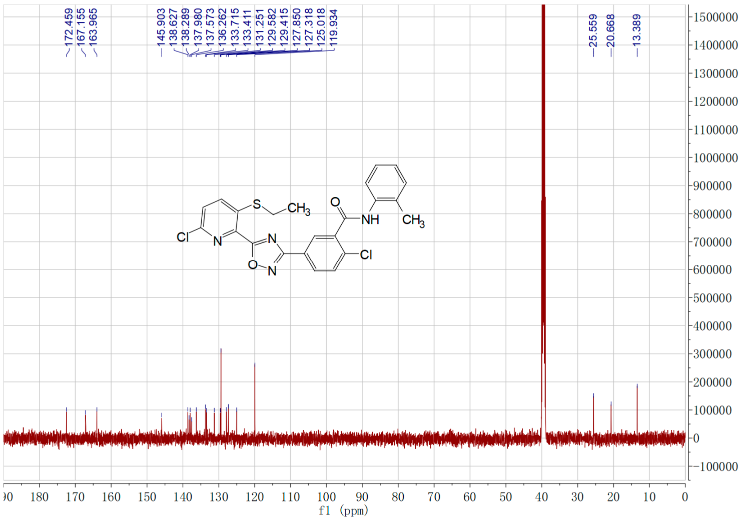

Fig. S5. The ^{13}C NMR spectrum of compound **9b**

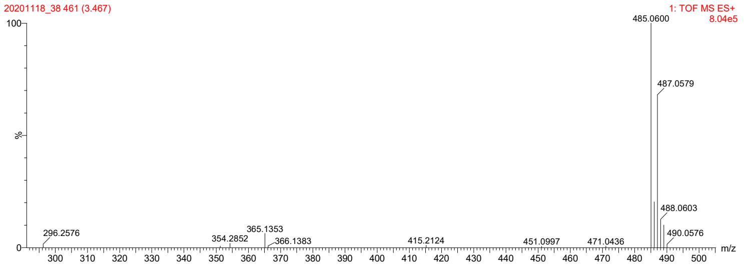

Fig. S6. The HRMS spectrum of compound **9b**

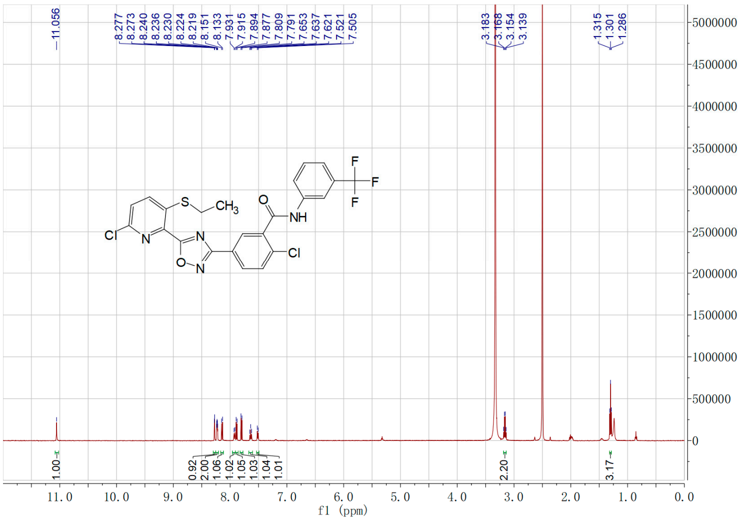

Fig. S7. The ^{1}H NMR spectrum of compound **9f**

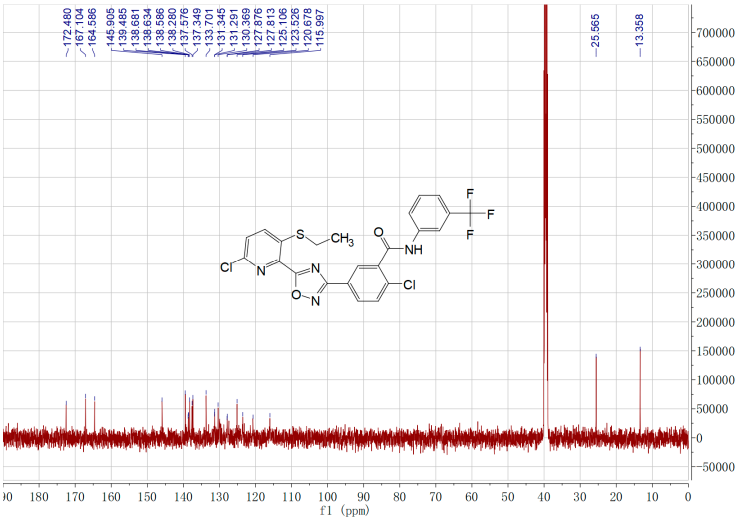

Fig. S8. The ^{13}C NMR spectrum of compound **9f**

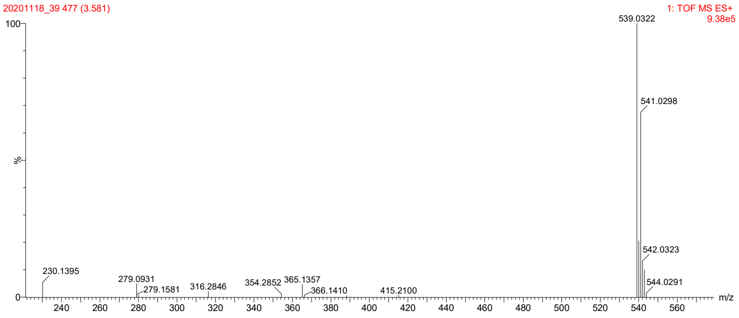

Fig. S9. The HRMS spectrum of compound **9f**

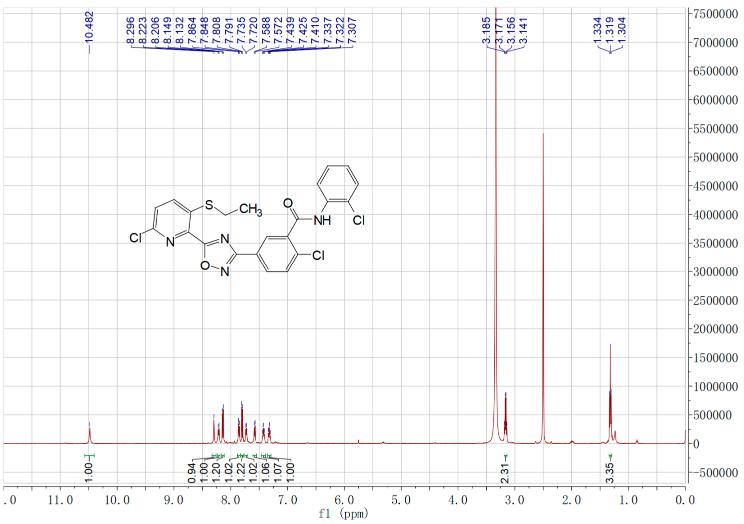

Fig. S10. The ^{1}H NMR spectrum of compound **9k**

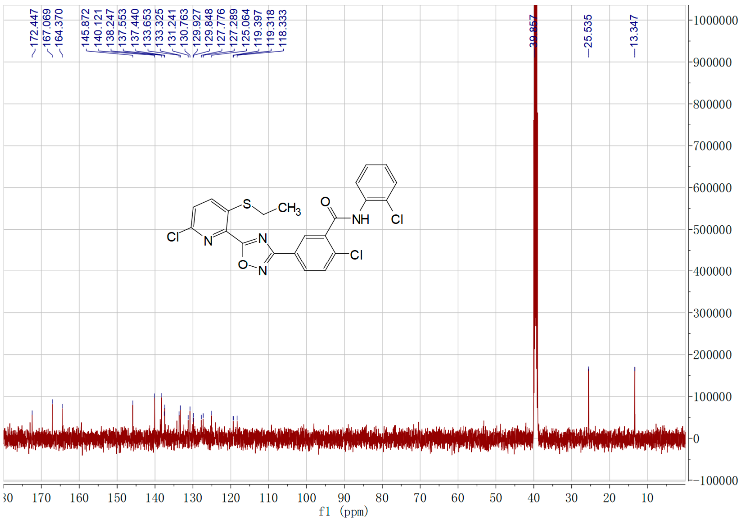

Fig. S11. The ^{13}C NMR spectrum of compound **9k**

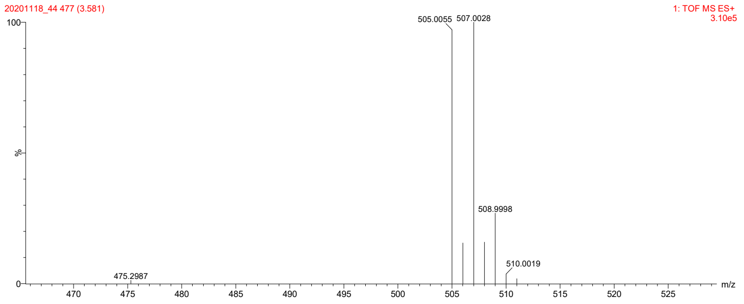

Fig. S12. The HRMS spectrum of compound **9k**

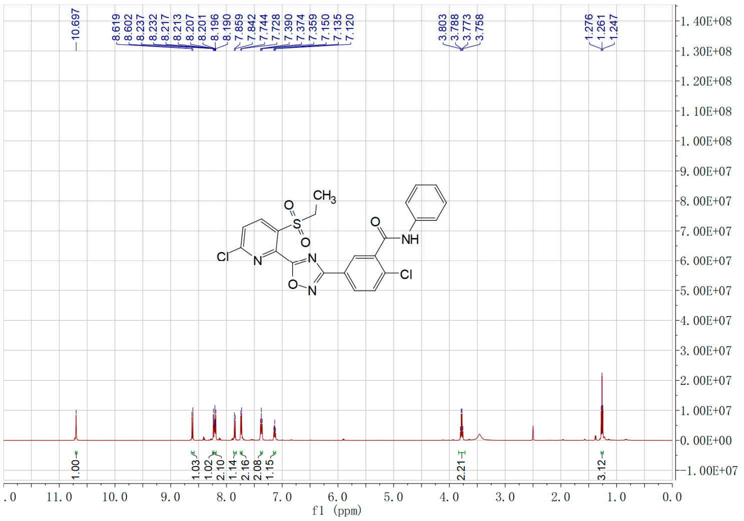

Fig. S13. The ^{1}H NMR spectrum of compound **10a**

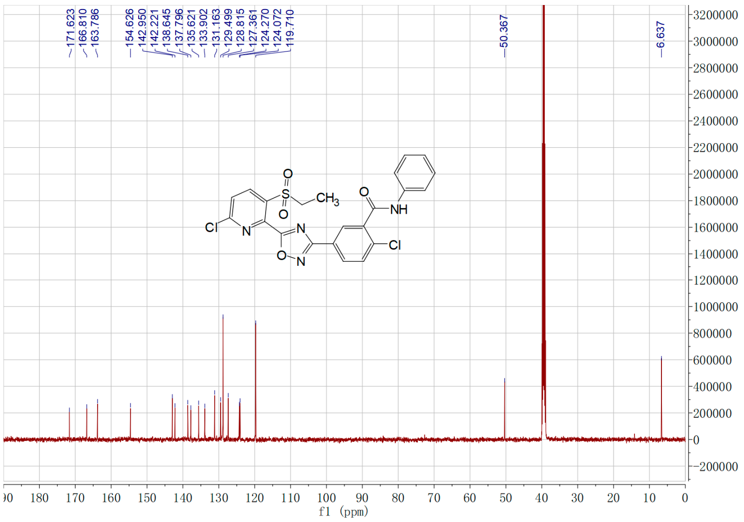

Fig. S14. The ^{13}C NMR spectrum of compound **10a**

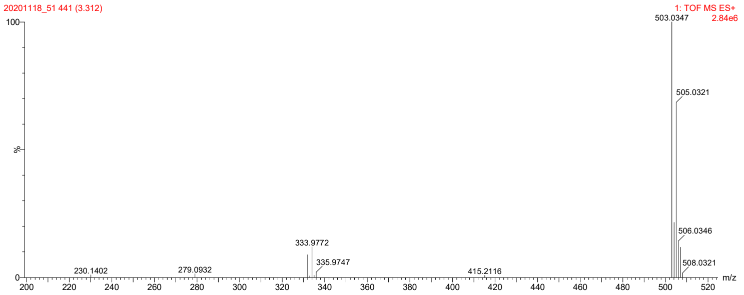

Fig. S15. The HRMS spectrum of compound **10a**

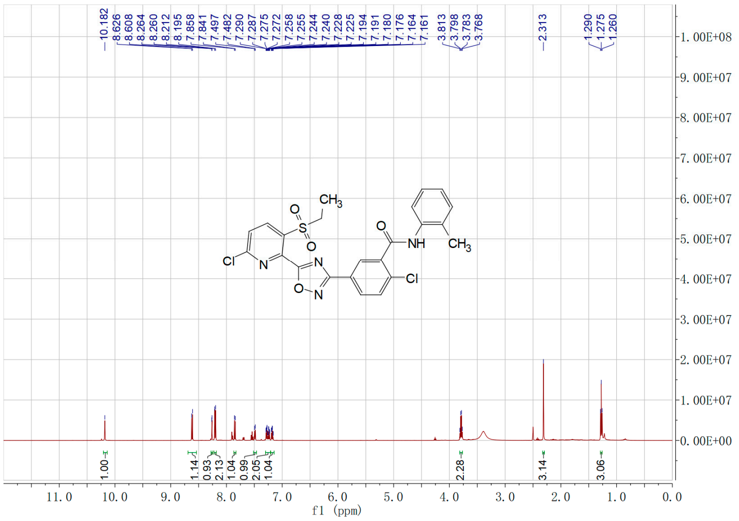

Fig. S16. The ^{1}H NMR spectrum of compound **10b**

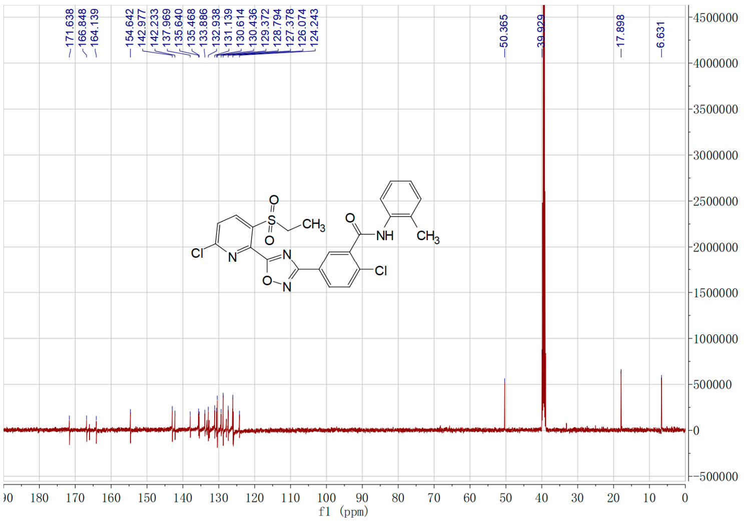

Fig. S17. The ^{13}C NMR spectrum of compound **10b**

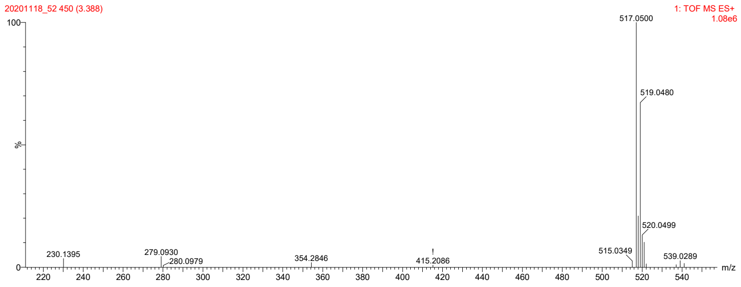

Fig. S18. The HRMS spectrum of compound **10b**

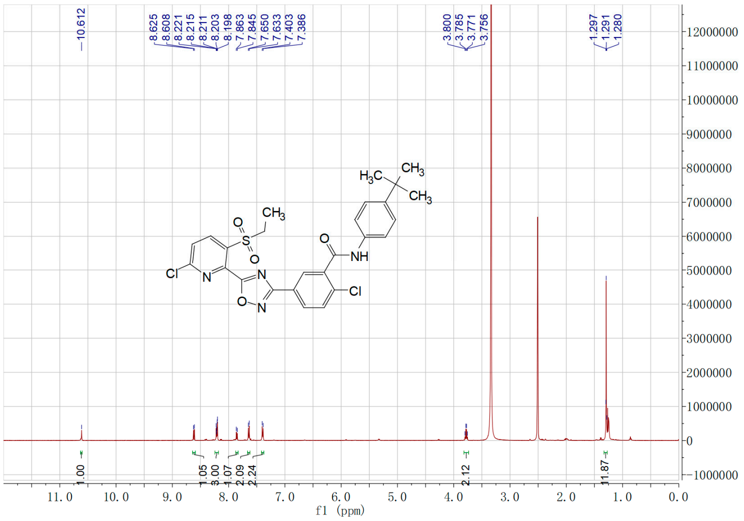

Fig. S19. The ^{1}H NMR spectrum of compound **10d**

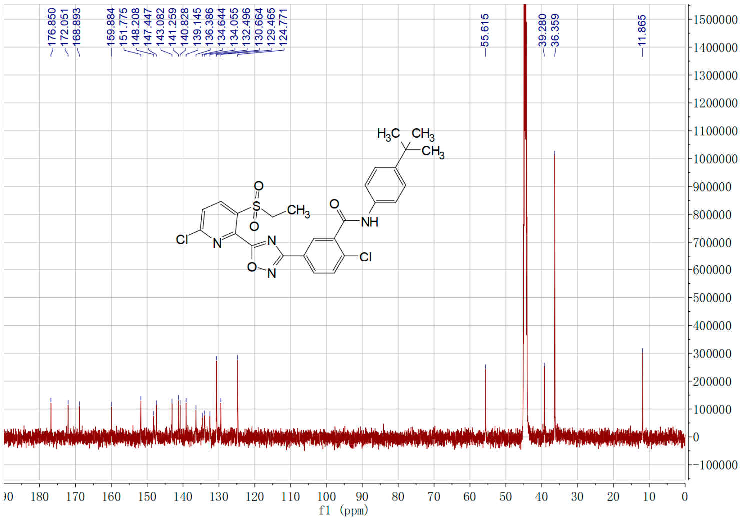

Fig. S20. The ^{13}C NMR spectrum of compound **10d**

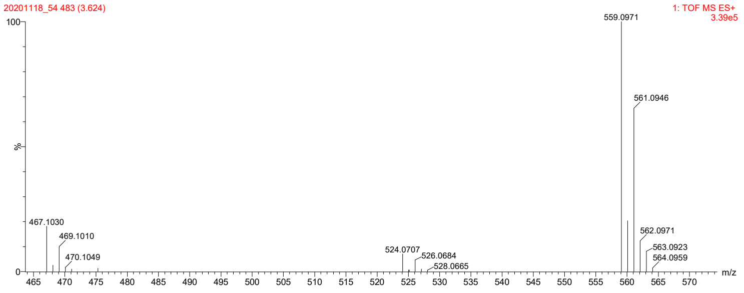

Fig. S21. The HRMS spectrum of compound **10d**

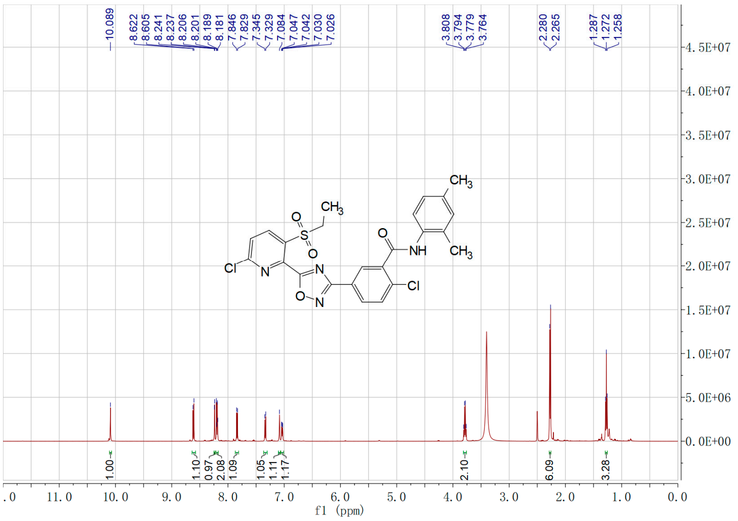

Fig. S22. The ^{1}H NMR spectrum of compound **10e**

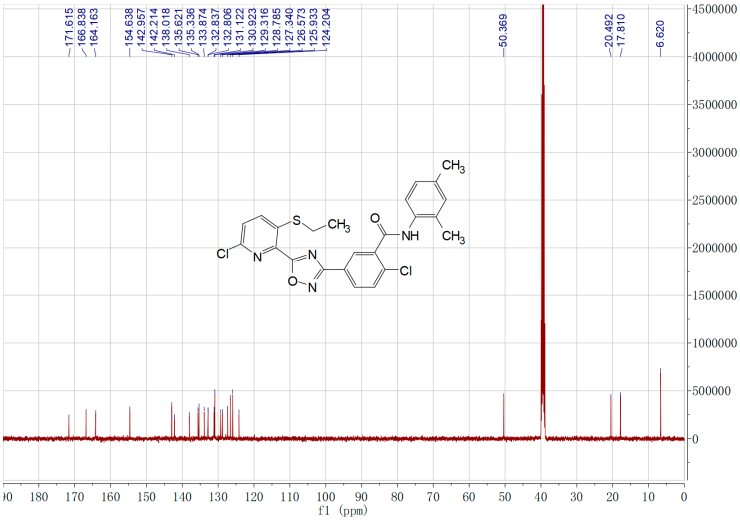

Fig. S23. The ^{13}C NMR spectrum of compound **10e**

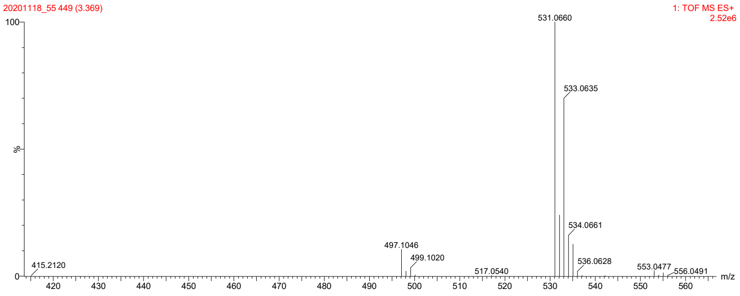

Fig. S24. The HRMS spectrum of compound **10e**

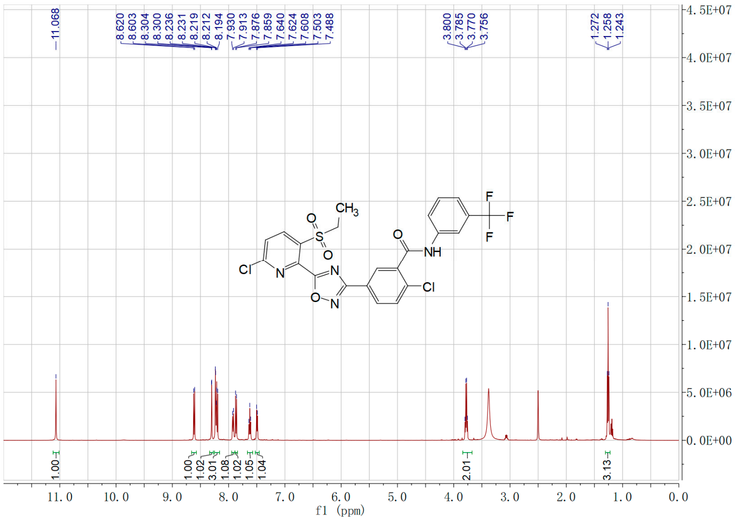

Fig. S25. The ^{1}H NMR spectrum of compound **10f**

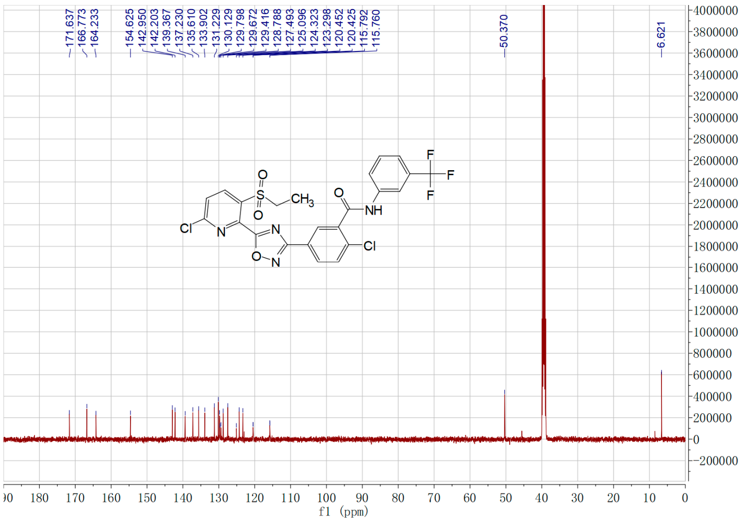

Fig. S26. The ^{13}C NMR spectrum of compound **10f**

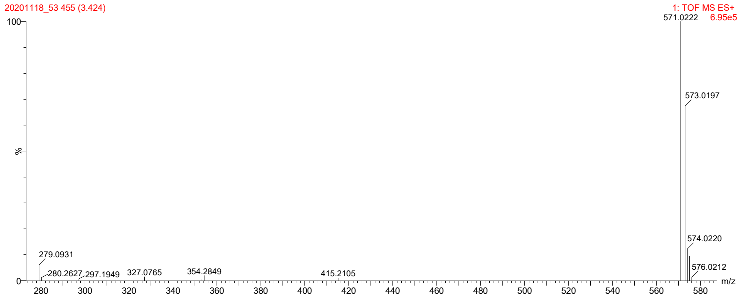

Fig. S27. The HRMS spectrum of compound **10f**

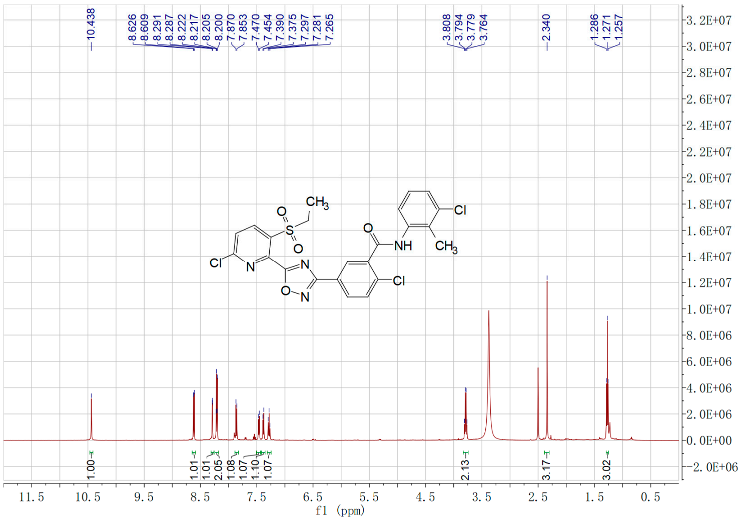

Fig. S28. The ^{1}H NMR spectrum of compound **10g**

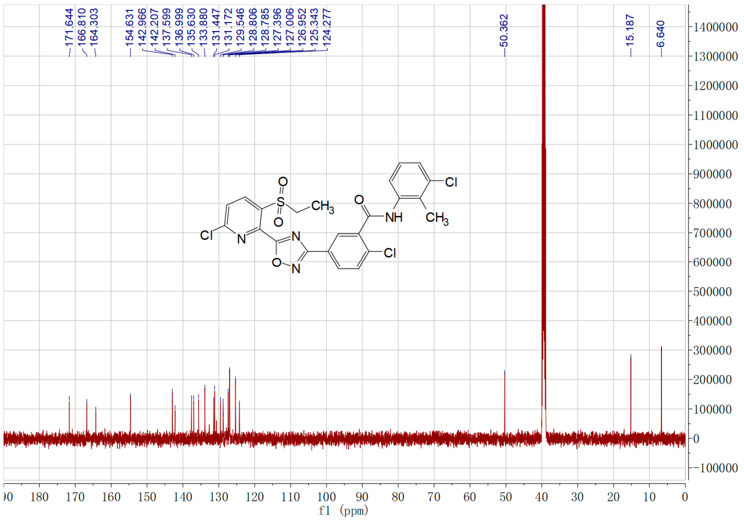

Fig. S29. The ^{13}C NMR spectrum of compound 10g

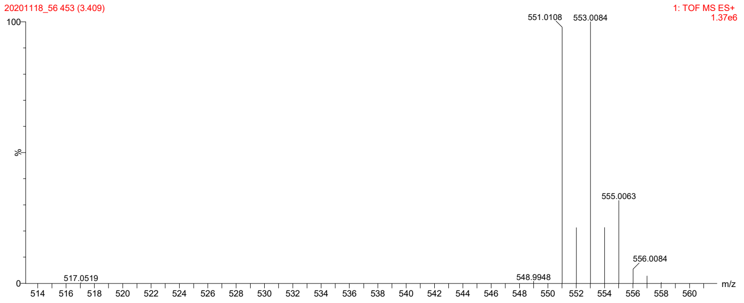

Fig. S30. The HRMS spectrum of compound **10g**

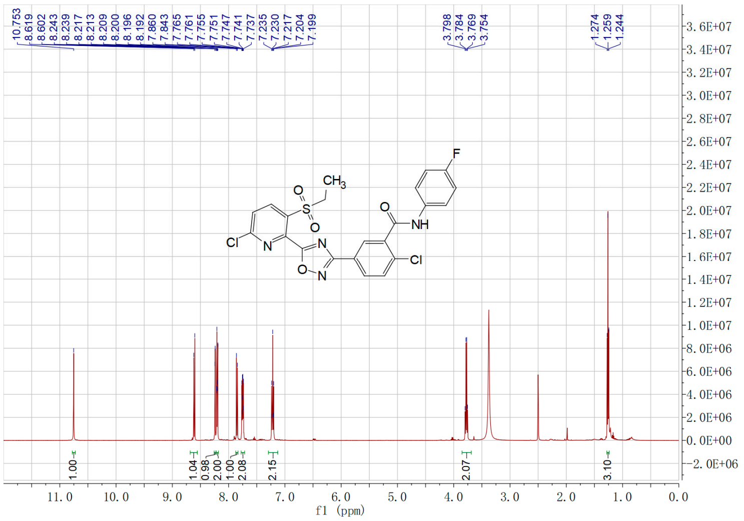

Fig. S31. The ^{1}H NMR spectrum of compound **10i**

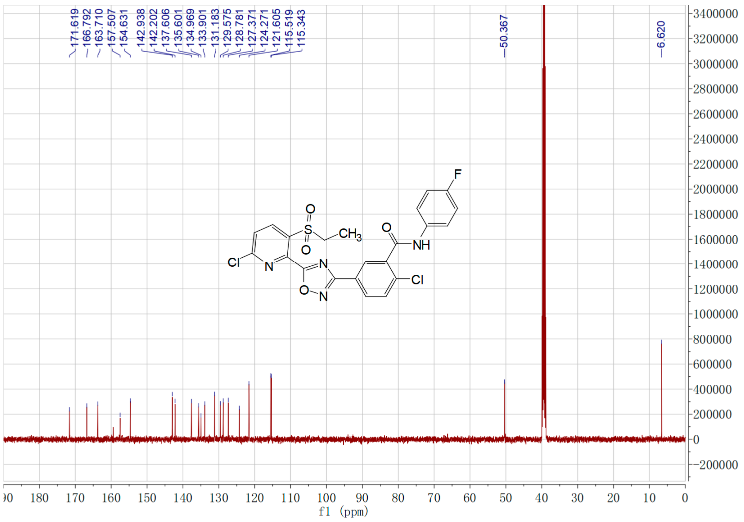

Fig. S32. The ^{13}C NMR spectrum of compound **10i**

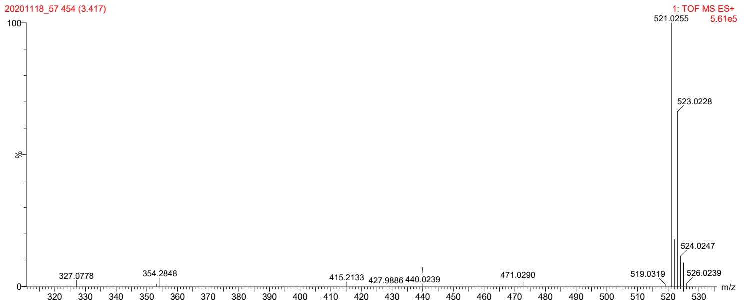

Fig. S33. The HRMS spectrum of compound **10i**

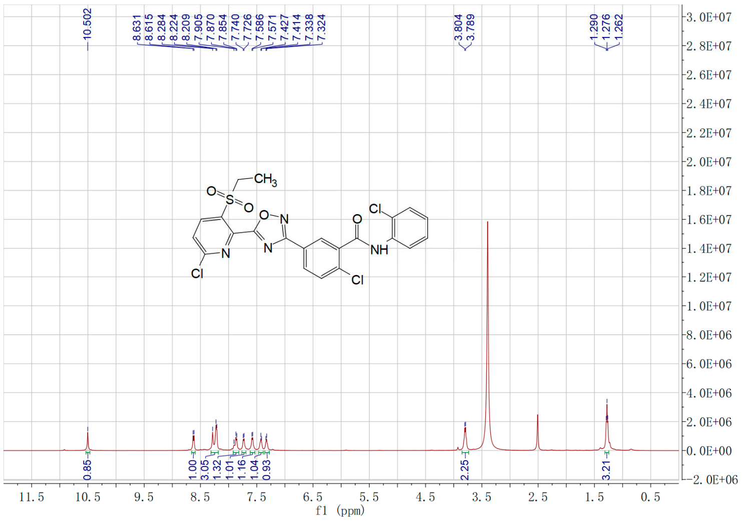

Fig. S34. The ^{1}H NMR spectrum of compound **10k**

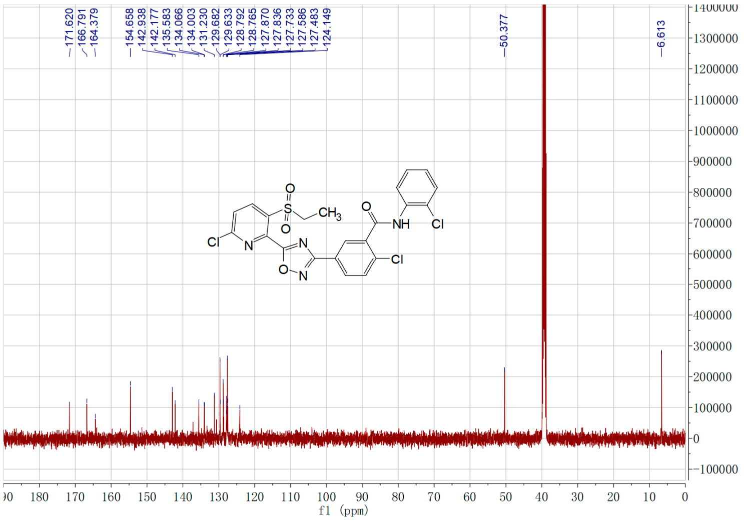

Fig. S35. The ^{13}C NMR spectrum of compound **10k**

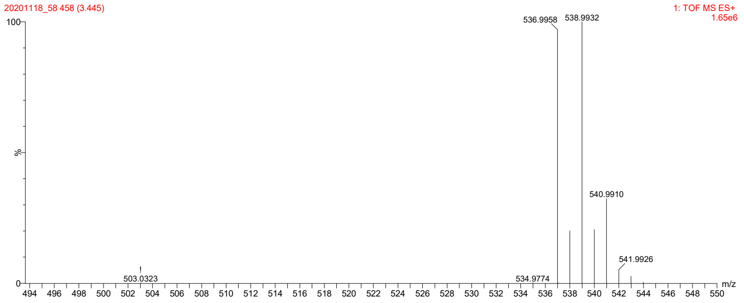

Fig. S36. The HRMS spectrum of compound **10k**

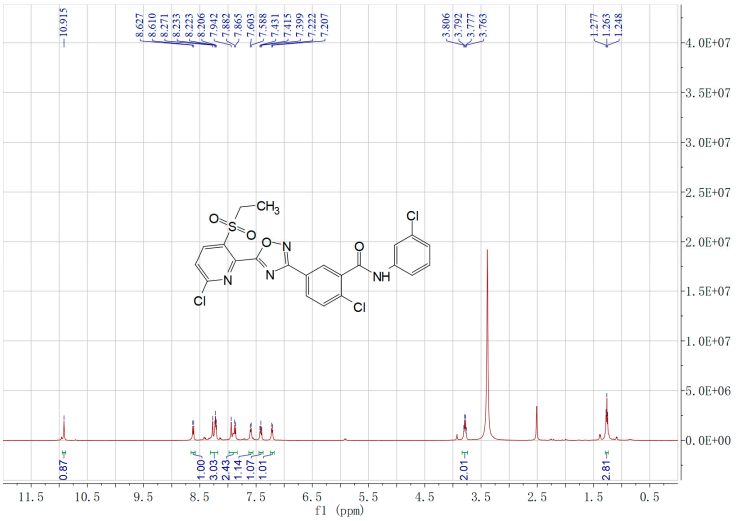

Fig. S37. The ^{1}H NMR spectrum of compound **10l**

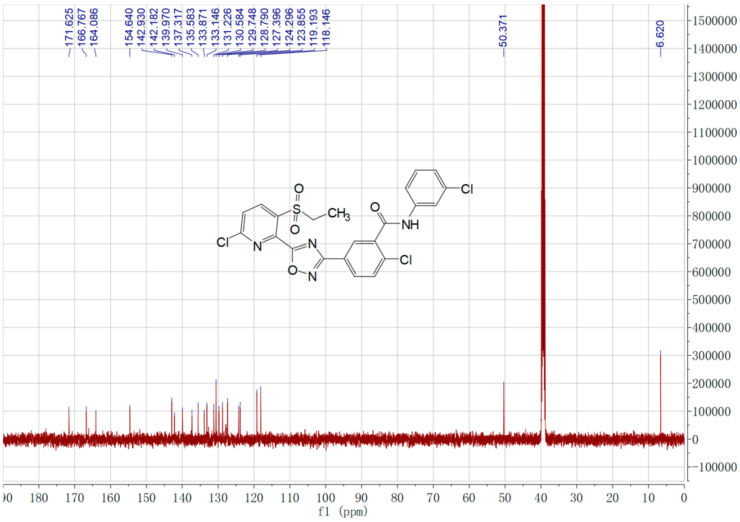

Fig. S38. The ^{13}C NMR spectrum of compound **10l**

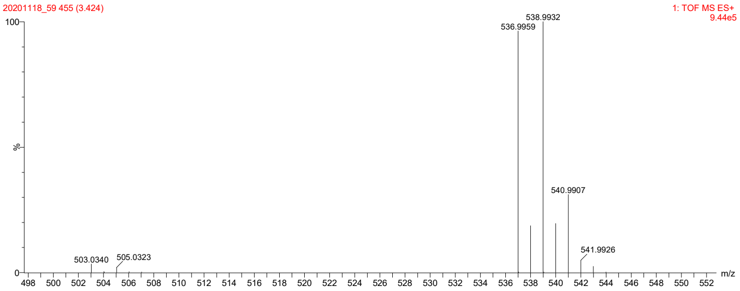

Fig. S39. The HRMS spectrum of compound **10l**
